# Effect of Demineralization Time on the Release of Bone Morphogenetic Protein in Indigenously Prepared Demineralized Freeze-Dried Bone Allografts: A Comparative In Vitro Study

**DOI:** 10.7759/cureus.84037

**Published:** 2025-05-13

**Authors:** Nasreen Ansari, Farrukh Faraz, Arundeep K Lamba, Shruti Tandon, Zainab Kamal, Zaid K Madni

**Affiliations:** 1 Periodontics, Maulana Azad Institute of Dental Sciences, New Delhi, IND; 2 Structural Immunology, International Centre for Genetic Engineering and Biotechnology, New Delhi, IND

**Keywords:** bone morphogenetic protein, demineralization time, demineralized freeze-dried bone allografts, osteoinductivity, tissue bank

## Abstract

Background: Demineralized freeze-dried bone allografts (DFDBAs) have emerged as a valuable biomaterial for regenerative therapy owing to their osteoinductive properties, which are attributable to the presence of bone morphogenetic proteins (BMPs). The different demineralization protocols used during graft processing by various tissue banks affect the levels of these growth factors. This study investigated the release of BMP-7 from a DFDBA prepared using two different demineralization time intervals.

Method: A total of 144 bone graft samples were prepared using a standard protocol. The samples were divided into two groups, each containing 72. Group 1 samples were demineralized for 18 hours, and Group 2 samples were demineralized for 90 minutes. All samples were gamma-irradiated and subjected to the BMP-7 extraction procedure. Quantification of BMP-7 was done using enzyme-linked immunosorbent assay (ELISA).

Results: The mean BMP-7 concentration of Group 1 was 135.609±11.943 ng/g and that of Group 2 was 95.442±9.226 ng/g. This difference between the mean BMP-7 concentration was found to be statistically significant (p = 0.013).

Conclusion: The study evaluated the effect of demineralization time on BMP-7 levels in indigenously prepared DFDBAs. By unveiling the relationship between demineralization time and BMP content, this study paves the way for optimizing DFDBA quality and advancing its potential in regenerative therapy.

## Introduction

Demineralized freeze-dried bone allografts (DFDBAs) as osteoinductive bone grafting material have been the cornerstone of research over the past few decades. DFDBA elicits its osteoinductive effect due to the acid demineralization step involved in graft processing, which exposes growth factors such as bone morphogenetic proteins (BMPs) [1,2].

BMPs are signaling molecules of the transforming growth factor-β (TGF-β) superfamily of proteins, which are critical in stimulating cell proliferation, angiogenesis, and osteogenesis, all of which contribute to the graft material's regenerative capacity. Among the 20 types of BMPs identified so far, only BMP-2, 4, 6, 7, and 9 have been shown to have significant osteogenic properties [3]. The formation of new bone modulated by BMPs and other associated growth factors is termed osteoinduction, where mesenchymal stem cells are directed to form cells of osteogenic lineage [4]. However, the inconsistent predictability seen in the DFDBA results when used in periodontal regeneration has raised questions about the processing methods used. Evidence in the literature indicates the potential reduction of BMPs and growth factors during the various stages of graft processing [5,6].

Various demineralization protocols are employed by tissue banks to process and prepare DFDBA. The most commonly used method involves treating allograft bone with 0.25 N or 0.5 N HCl to effectively remove minerals while preserving BMP content. A standard ratio of 1 g of bone per 50 ml of HCl is typically used for optimal demineralization [7]. However, there is no defined protocol that exists for the time duration for which the graft is exposed to acid, with reported time intervals ranging from five minutes to 24 hours. In this study, we selected 90 minutes and 18 hours based on existing evidence. Research indicates that extractable BMP-7 content is highest at 90 minutes, while the 18-hour duration aligns with the protocol followed by the Central Tissue Bank at Maulana Azad Institute of Dental Sciences, New Delhi [8,9].

Considering all these factors, this study was conducted to examine the variability in BMP-7 levels in indigenously prepared graft samples under two different demineralization time durations, with the objective of creating allografts that are effective, safe, and cost-efficient while adhering to internationally recognized standards.

## Materials and methods

All procedures were performed in compliance with ethical standards and after obtaining the approval of the institutional ethical committee. This study involved the preparation of 144 DFDBA from eight donors undergoing hip arthroplasty at the Department of Orthopaedics, Lok Nayak Hospital, New Delhi. Prior to surgery, serological tests for HIV/AIDS, hepatitis B and C, and syphilis were conducted. Patients aged over 70, along with those with a history of infection, prior irradiation at the donor site, or connective tissue disorders, were excluded from the study.

After obtaining femur heads from the operation theatre, they were immediately placed in 0.9% saline solution in sterile plastic packaging and were transported to the Central Tissue Bank, Department of Periodontology, Maulana Azad Institute of Dental Sciences (MAIDS), in a temperature-controlled storage box maintained at 4°C for storage and processing.

**Figure 1:**
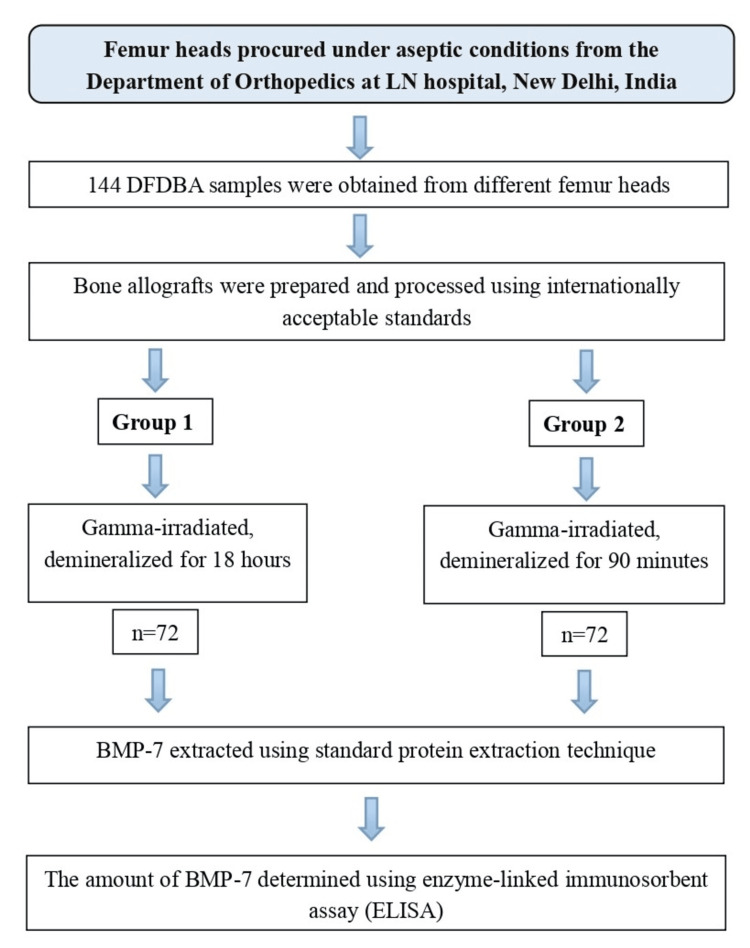
Study design DFDBA: demineralized freeze-dried bone allograft; LN: Lok Nayak Hospital; BMP: bone morphogenetic protein

The 144 DFDBA samples were prepared from eight femur heads collected from eight donors (four males and four females) aged 33-55 years. The sample size estimation was conducted using G*Power software version 3.1 (Heinrich Heine University Düsseldorf, Düsseldorf, Germany), considering data from previous studies, and samples were randomly assigned to two groups (Group 1 and Group 2) using a computer-generated allocation called Research Randomizer (https://www.randomizer.org), with 72 samples in each group. Group 1 samples were demineralized for 18 hours, and Group 2 samples were demineralized for 90 minutes. Each sample was analyzed for BMP-7 levels, and the results were evaluated and compared. The study design is shown in Figure 1.

**Figure 2:**
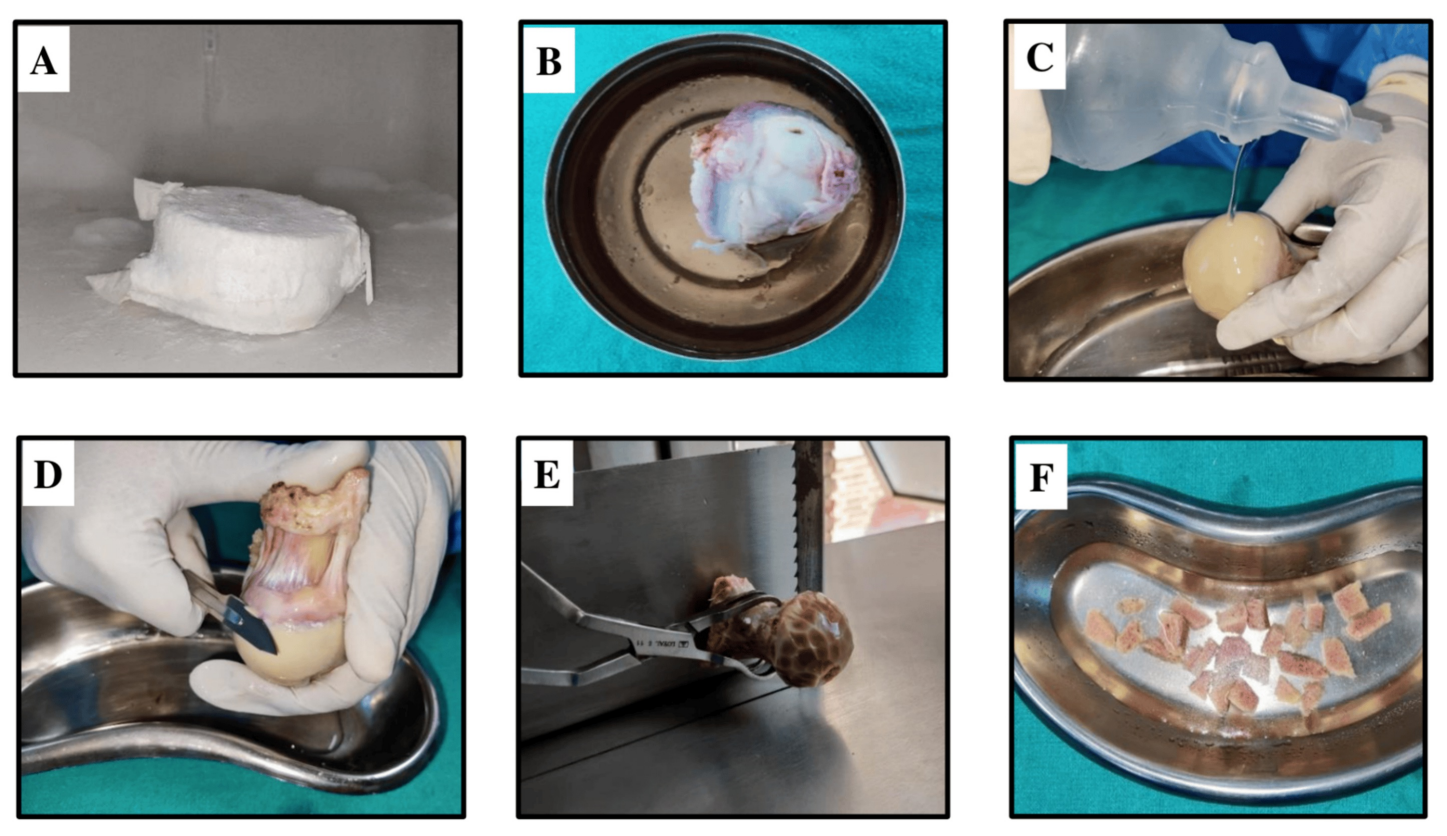
Processing of demineralized freeze-dried bone allograft (A) Femur head in a deep freezer under sterile conditions. (B) Thawed femur head. (C) Washing step with saline. (D) Removal of soft tissue and cartilage. (E, F) Cutting the femur head into small blocks with a bone cutter

The processing of DFDBA was done according to internationally acceptable standards [9]. Procured femur heads were stored at -80°C in a deep freezer at the Central Tissue Bank, MAIDS, for one month. This ensures a complete reduction of antigenicity of allograft bone [10]. For processing, stored femoral heads were thawed, cleaned of cartilage and soft tissues, and cut into small cortico-cancellous pieces using a bone cutter (ADE, Robusta S023, Hamburg, Germany), as shown in Figure 2.

**Figure 3:**
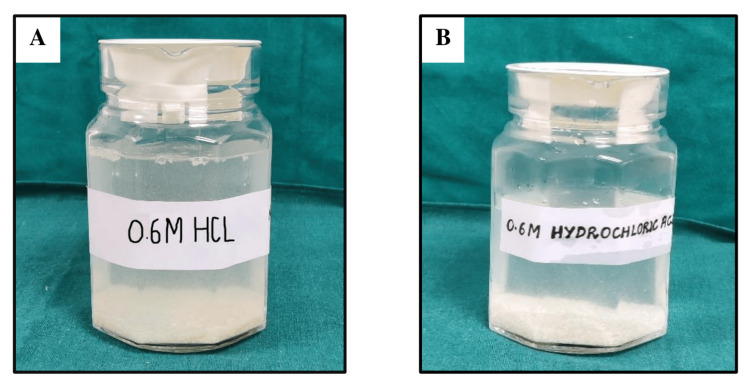
Demineralization process (A) Graft demineralized for 18 hours. (B) Graft demineralized for 90 minutes

The bone pieces were thoroughly cleaned using an ultrasonic bath (Water Bath Shaker, Tanco, New Delhi, India) and a centrifuge machine (REMI R-8C Centrifuge, Mumbai, India) to eliminate blood and debris. Following the cleaning, the defatting procedure was done using an alcohol-chloroform solution (ratio 1:1). Once the defatting procedure was completed, lyophilization was done. The bone pieces were ground into fine particles using a bone mill, and the bone powder of the desired particle size (500-1000 µm) was collected using different mesh combined stainless steel sieves (Henan Zhongrentian Machinery Manufacturing Co. Ltd., Zhengzhou, China). For demineralization, the grafts were divided into two groups. Group 1 and Group 2 samples were kept in 0.6 M HCl for 18 hours and 90 minutes, respectively, at 25°C. A standard ratio of 1 g of bone per 50 ml of HCl was used for optimal demineralization. The demineralization procedure at two different time durations is shown in Figure 3.

**Figure 4:**
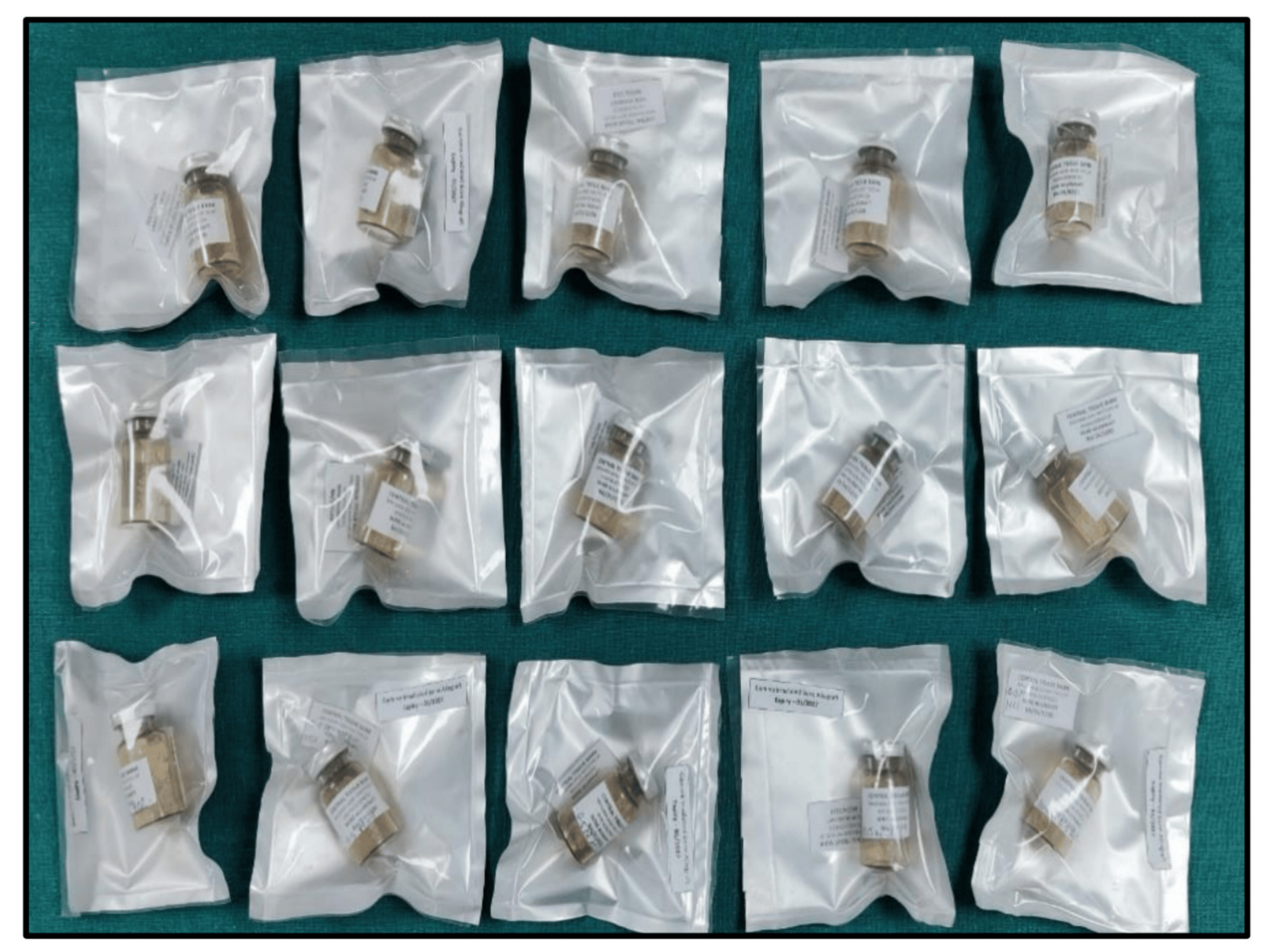
Prepared DFDBAs sealed and packed under sterile conditions DFDBAs: demineralized freeze-dried bone allografts

Subsequently, the cleaning process was repeated to eliminate any remaining acid residues. The processed graft particles were transferred into sterile borosilicate vials and subjected to lyophilization in a freeze-dryer (Labconco Triad System, Kansas City, Missouri). The samples were sealed and packed using a double-sided low-density polyethylene sheet under sterile conditions, as shown in Figure 4.

All samples were sent for gamma irradiation at Shriram Institute of Industrial Research with a 25 kGy dose as per international standards (ISO-13409: 2002). All DFDBA samples were subjected to the BMP extraction procedure at the Structural Immunological Lab, International Centre for Genetic Engineering and Biotechnology (ICGEB), New Delhi, according to a standard protein extraction technique [11]. The extract collected from Group 1 and Group 2 samples was subjected to ELISA (enzyme-linked immunosorbent assay) testing to quantify the amount of BMP-7 using the Human BMP-7 ELISA kit (G-Biosciences, St. Louis, MO, USA).

## Results

This study aimed to evaluate and compare the mean BMP-7 concentrations of 144 indigenously produced DFDBA samples subjected to two different demineralization time intervals. The samples were divided into two groups: Group 1, demineralized for 18 hours, and Group 2, demineralized for 90 minutes, with 72 samples in each group.

The 144 DFDBA samples were prepared from eight femur heads obtained from eight donors (four males and four females) aged 33 to 55 years. A simple random sampling technique was employed to assign samples to the two groups, with the allocation sequence generated using computer software (Research Randomizer). All DFDBA samples underwent the BMP extraction procedure, and the extracts collected from Group 1 and Group 2 were analyzed using ELISA to quantify BMP-7 levels.

For statistical analysis, data normality was assessed using the Shapiro-Wilk test, confirming a normal distribution. Group comparisons were conducted using an independent t-test, with statistical significance defined as a p-value of less than 0.05. The mean BMP-7 concentration in Group 1 (demineralized for 18 hours) was 135.609 ± 11.943 ng/g, while Group 2 (demineralized for 90 minutes) had a mean concentration of 95.442 ± 9.226 ng/g. This difference was statistically significant (p = 0.013), as presented in Table 1.

**Table 1:** Comparison of the mean BMP-7 levels of DFDBA samples based on demineralization time * Statistically significant SD: standard deviation; BMP: bone morphogenetic protein; DFDBA: demineralized freeze-dried bone allograft

Type of comparison		Mean BMP-7 ± SD (ng/g)	p-value
Comparison of mean BMP-7 levels of samples demineralized for 18 hours and 90 minutes	Group 1	135.609 ± 11.943	0.013*
	Group 2	95.442 ± 9.226	

## Discussion

The utilization of bone allografts in regenerative medicine has gathered significant attention due to their pivotal role in inducing bone formation and tissue regeneration. Autogenous grafts have all the qualities of the ideal bone grafting material, but their scarcity and associated donor site morbidity prevent their widespread use. Compared to xenografts and other synthetic biomaterials that are only osteoconductive, bone allografts are considered superior because they address these drawbacks [12]. Despite these benefits, numerous studies in the literature show variable results when bone allografts are used for periodontal regenerative therapy, questioning the effectiveness of these graft materials [13]. Possible explanations accounting for this variation in clinical results could be due to various factors, such as the age of the donor, particle size and shape, method of processing of grafts, and methods of sterilization used [14-16].

As demineralization time is an important determinant that may influence the clinical outcome of DFDBA, we correlated the effect of different demineralization time intervals used during graft processing and examined the amount of extractable BMP-7 obtained from two groups. In the present study, DFDBA samples were prepared using two different demineralization protocols, and BMP-7 was extracted using guanidine HCl, which is proven to be the most efficient among other methods of protein extraction [17]. Bone allograft samples were prepared using a demineralization time of 18 hours and 90 minutes, and BMP-7 was found to be significantly higher in grafts demineralized for 18 hours (Group 1) compared to those demineralized for 90 minutes (Group 2). This disparity between the two groups may be attributed to the inefficient demineralization of allograft bone at 90 minutes, leading to less BMP release in the extraction buffer.

It has been reported that both short (0 to 45 minutes) and longer durations (24 hours) of acid exposure to grafts reduce the measurable BMP-7 levels due to the inability of exposure of matrix-associated BMPs and diffusional loss due to prolonged acid exposure, respectively [18]. Zhang et al. (1997) reported reduced osteoinductive potential of the bone matrix subjected to a demineralization time of 90 minutes compared to those demineralized for 24 hours in animal models. They proposed that allograft bone be demineralized until the residual calcium becomes approximately 2%. This is important, as 2% residual calcium acts as nucleation sites for the redeposition of calcium phosphate salts and aids in bone deposition at grafted sites [19]. Koga et al. (2016) reported superior bone regenerative activity within animal models with the use of partially demineralized dentin matrix compared to a completely demineralized dentin matrix in the early stage of bone regeneration. They described that cell morphology is sensitive to surface nanostructure, as almost no osteoblasts were seen attached to the surface of less demineralized dentin matrix, while many osteoblasts were attached and spread on the surface of demineralized dentin matrix, as demonstrated in electron-microscopic observation [20].

By correlating these findings, our study underscores the significance of determining the appropriate duration for allograft demineralization. Optimizing acid exposure time, combined with accurate monitoring of residual calcium levels in the demineralized graft, can produce superior allografts with enhanced BMP bioavailability essential for regenerative procedures.

Once the processing steps for allografts are completed, it is important to ensure that the graft is rendered completely sterile to prevent allograft-associated infections. In the present study, all the prepared grafts were gamma-irradiated. A dose of 25 kGy was selected as per the standards of the International Atomic Energy Agency (IAEA 2002), American Association of Tissue Banking (AATB 2002), and European Association of Musculo-Skeletal Transplantation (EAMST 2005) [21]. To meet the increasing requirement of regenerative biomaterials in clinical practice and to surpass the high cost of commercially available bone allografts, DFDBA are indigenously prepared and processed at Central Tissue Bank, MAIDS, New Delhi, India, as per internationally acceptable standards [22-25].

Despite careful study design and methodology, a few limitations exist in the present study that should be taken into account for future research and clinical applications. Since the BMP was extracted from DFDBA in a controlled laboratory setting, it may not fully replicate the biological environment of in vivo conditions. Hence, the actual clinical performance of the grafts could vary when used in patients.

While this study establishes a foundation for improving DFDBA quality by emphasizing the crucial relationship between demineralization time and BMP content, other factors such as donor age, gender, sterilization methods, and biological variations may also impact BMP-7 levels in prepared allografts [26,27]. These factors should be carefully considered during graft processing in tissue banks. Further research with a larger sample size is needed to better understand their influence.

Among the various osteoinductive proteins, BMP-7 is found in higher concentrations within the bone matrix, making it the preferred marker for this study [28]. However, other growth factors (BMP-2, 4, 6, and 9) in DFDBA may also significantly contribute to graft effectiveness. Therefore, further research incorporating additional osteoinductive proteins is necessary to gain a more comprehensive understanding of their role.

## Conclusions

Within the limitations of this study, it can be concluded that extended demineralization time increases the bioavailability of BMPs. This is demonstrated by the significantly higher BMP-7 concentration in grafts demineralized for 18 hours compared to 90 minutes. Shorter demineralization durations may result in inadequate exposure of matrix-associated BMPs, potentially compromising graft effectiveness.

Ensuring the grafts are made using the correct processing protocols under stringently sterile conditions could resolve the problem of limited availability and the expensive cost of biomaterial for periodontal regeneration. However, further studies with DFDBA prepared using different demineralization protocols are required to formulate concrete evidence.
